# Supplementary figures and images for: Dose-dependent thresholds of dexamethasone destabilize CAR T-cell treatment efficacy
Source: PLoS Comput Biol. 2022 Jan 26;18(1):e1009504. doi: 10.1371/journal.pcbi.1009504 (PMC8820647; doi:10.1371/journal.pcbi.1009504)

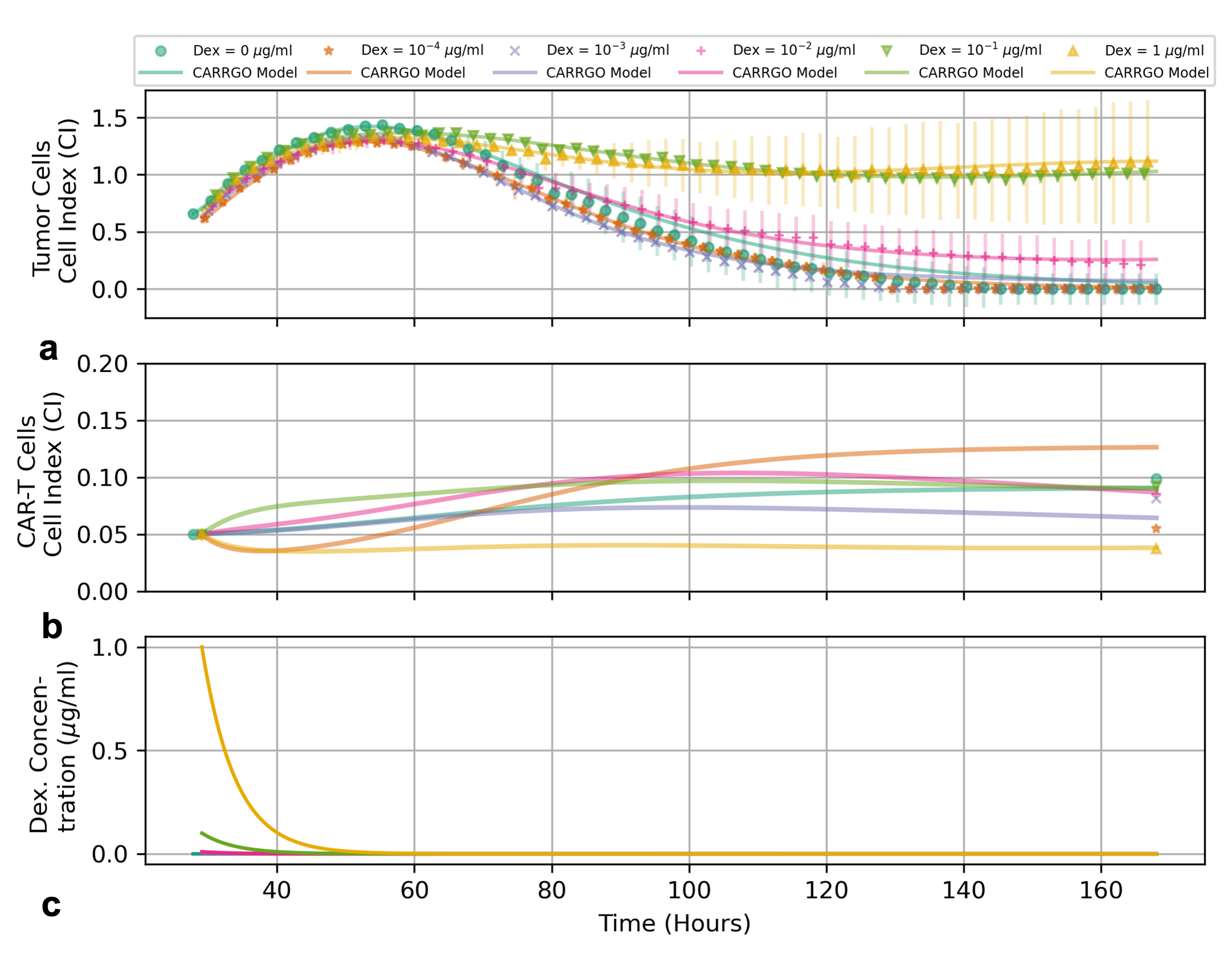

Supplement: S1 Fig — Graphs of tumor cells, CAR T-cells, and Dex concentration over time for tumor cell line PBT138 with an initial effector to target ratio of 1:4. Temporal measurements of tumor cell population and the initial and final CAR T-cell measurements are represented by symbols, and CARRGO model predictions are represented by lines. Colors and symbol types vary to reflect initial Dex concentrations (see top legend). The progression of the tumor cell curves as initial Dex concentration increases demonstrate the effect of Dex to reduce CAR T-cell efficacy. In particular, CAR T-cell treatment is successful at low Dex initial Dex concentrations (0, 10−4, and 10−3 μg/ml) and fails at higher initial Dex concentrations (10−2, 10−1, and 1 μg/ml), resulting in tumor cell progression. Experimental measurements for the tumor cell population are down-sampled by 1/10 for clarity. (TIF) [file pcbi.1009504.s002.tif]

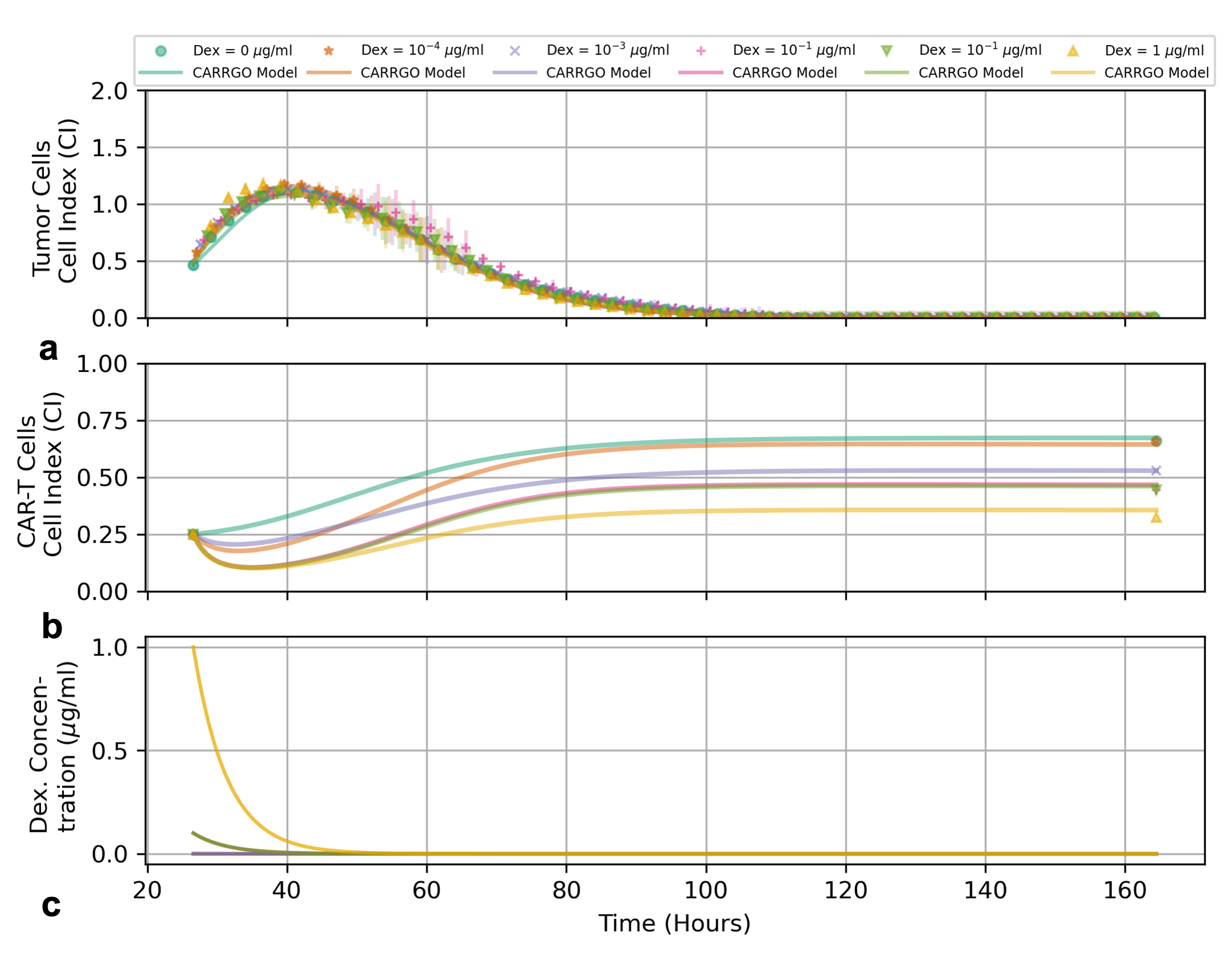

Supplement: S2 Fig — Similar graphical information as S1 Fig presented for tumor cell line PBT030 with an initial effector-to-target ratio of 1:4. For all initial Dex concentrations, treatment success is observed, resulting in complete tumor death. (TIF) [file pcbi.1009504.s003.tif]

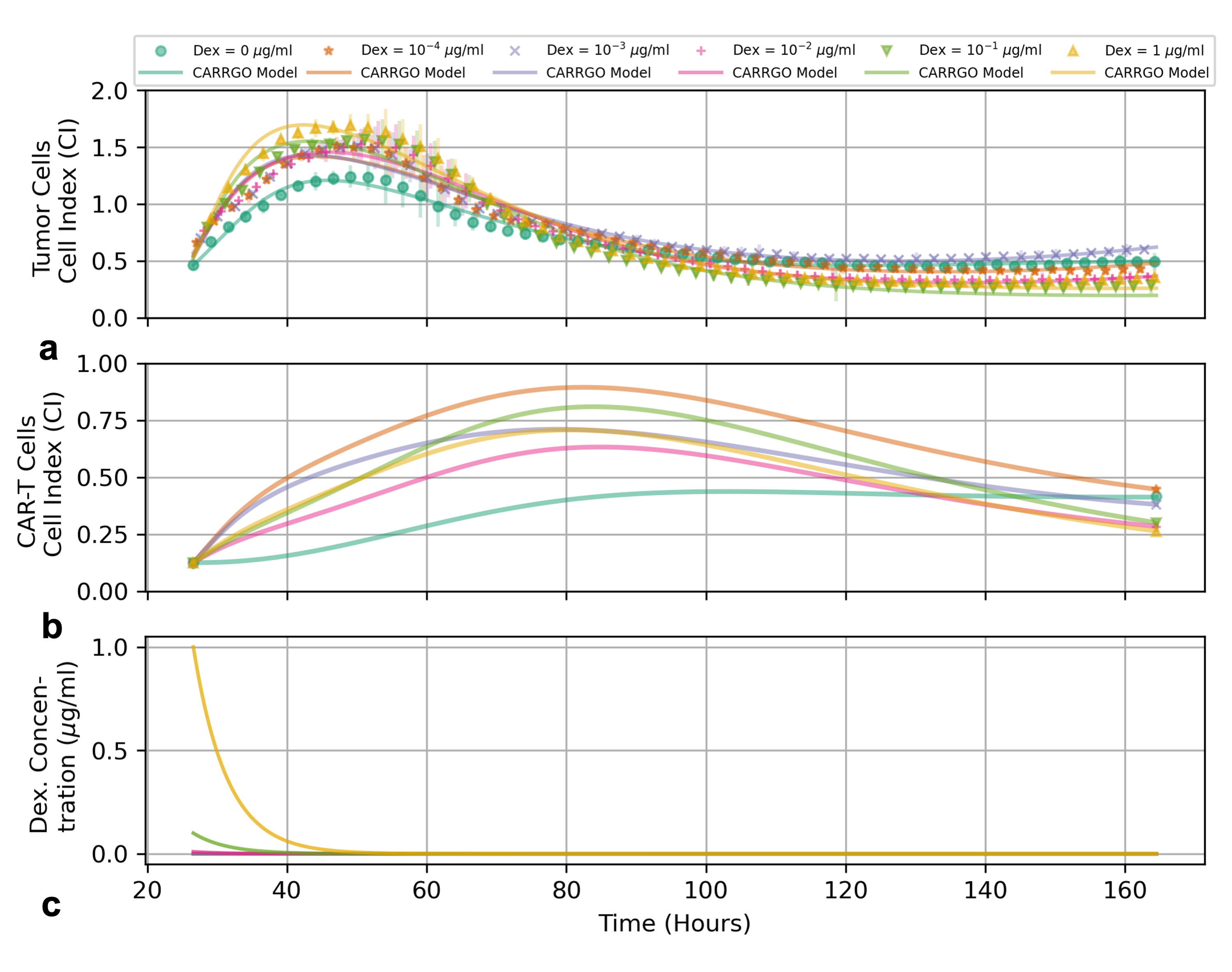

Supplement: S3 Fig — Similar graphical information as S1 Fig presented for tumor cell line PBT030 with an initial effector-to-target ratio of 1:8. For all initial Dex concentrations, treatment failure is observed, resulting in tumor cell progression that generally increases with increasing initial Dex concentrations. (TIF) [file pcbi.1009504.s004.tif]

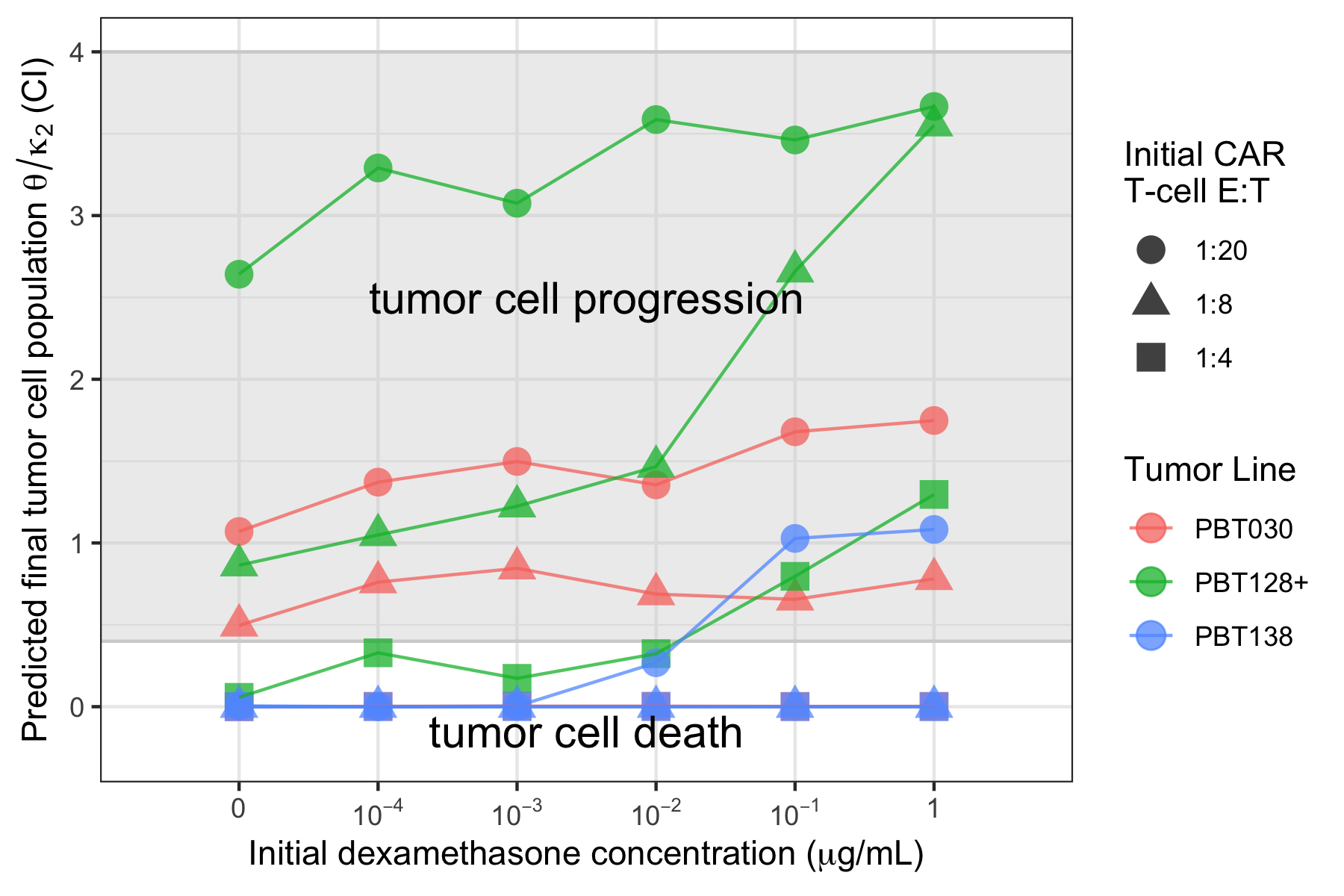

Supplement: S4 Fig — The ratio of CAR T-cell death (θ) to CAR T-cell proliferation/exhaustion (κ2) rates predict CAR T-cell treatment success (tumor cell death) or failure (tumor cell progression). We observed that a ratio of θ/κ2 ≈ 0.4 CI as the predicted final tumor cell population serves as a threshold for observed tumor progression or death. The threshold of θ/κ2 ≈ 0.4 CI was consistent across all three tumor cell lines (denoted by color), CAR T-cell E:T ratios (denoted by shape), and initial Dex concentrations (denoted by location along the horizontal axis). (TIF) [file pcbi.1009504.s005.tif]
